# Glucose-Dependent Effects of Exogenous Glucagon-Like Peptide-2 on Circulating Glucagon Levels in Healthy Men

**DOI:** 10.1210/jendso/bvaf215

**Published:** 2025-12-23

**Authors:** Nikolaj E Sørum, Tore Magnussen, Nina L Hansen, Casper K Nielsen, Jens J Holst, Bolette Hartmann, Kristine Henriksen, Joachim Størling, Jens Faber, Mikkel B Christensen, Asger B Lund, Filip K Knop

**Affiliations:** Center for Clinical Metabolic Research, Gentofte Hospital, University of Copenhagen, 2900 Hellerup, Denmark; Center for Clinical Metabolic Research, Gentofte Hospital, University of Copenhagen, 2900 Hellerup, Denmark; Center for Clinical Metabolic Research, Gentofte Hospital, University of Copenhagen, 2900 Hellerup, Denmark; Center for Clinical Metabolic Research, Gentofte Hospital, University of Copenhagen, 2900 Hellerup, Denmark; Department of Biomedical Sciences, Faculty of Health and Medical Sciences, University of Copenhagen, 2200 Copenhagen N, Denmark; Novo Nordisk Foundation Center for Basic Metabolic Research, Faculty of Health and Medical Sciences, University of Copenhagen, 2200 Copenhagen N, Denmark; Department of Biomedical Sciences, Faculty of Health and Medical Sciences, University of Copenhagen, 2200 Copenhagen N, Denmark; Steno Diabetes Center Copenhagen, Translational Type 1 Diabetes Research, 2730 Herlev, Denmark; Department of Biomedical Sciences, Faculty of Health and Medical Sciences, University of Copenhagen, 2200 Copenhagen N, Denmark; Steno Diabetes Center Copenhagen, Translational Type 1 Diabetes Research, 2730 Herlev, Denmark; Department of Clinical Medicine, Faculty of Health and Medical Sciences, University of Copenhagen, 2200 Copenhagen N, Denmark; Center for Clinical Metabolic Research, Gentofte Hospital, University of Copenhagen, 2900 Hellerup, Denmark; Department of Clinical Medicine, Faculty of Health and Medical Sciences, University of Copenhagen, 2200 Copenhagen N, Denmark; Department of Clinical Pharmacology, Copenhagen University Hospital–Bispebjerg and Frederiksberg, 2400 Copenhagen NV, Denmark; Center for Clinical Metabolic Research, Gentofte Hospital, University of Copenhagen, 2900 Hellerup, Denmark; Steno Diabetes Center Copenhagen, Translational Type 1 Diabetes Research, 2730 Herlev, Denmark; Department of Clinical Medicine, Faculty of Health and Medical Sciences, University of Copenhagen, 2200 Copenhagen N, Denmark; Center for Clinical Metabolic Research, Gentofte Hospital, University of Copenhagen, 2900 Hellerup, Denmark; Department of Clinical Medicine, Faculty of Health and Medical Sciences, University of Copenhagen, 2200 Copenhagen N, Denmark

**Keywords:** glucagon-like peptide 2, GLP-2, hypoglycemia, euglycemia, hyperglycemia, glucagon

## Abstract

**Context:**

Despite evidence of possible glucagonotropic effects, the role of the gut hormone glucagon-like peptide 2 (GLP-2) in glucose metabolism is unclear.

**Objective:**

This work aimed to evaluate the effect of exogenous GLP-2 on plasma glucagon levels during hypoglycemia, euglycemia, and hyperglycemia in healthy male volunteers.

**Methods:**

A randomized, double-blind, placebo-controlled, crossover study was conducted with supportive ex vivo human islet experiments. Participants included 10 lean, healthy men, median (interquartile range) aged 22 (21-23) years, with body mass index of 23.5 (23.3-23.8) and glycated hemoglobin A_1c_ of 4.8 (4.6-5.1)% (29 (26.5-32.5) mmol/mol). During 6 separate study days, GLP-2 (6 pmol/kg/min for 10 minutes and 2 pmol/kg/min for the following 90 minutes) and placebo (saline), respectively, were infused intravenously during insulin-induced hypoglycemia (∼2.5 mmol/L), euglycemia (∼5 mmol/L), or hyperglycemia (∼10 mmol/L). Primary outcome was baseline-subtracted area under the curve for plasma glucagon, and secondary outcomes were serum insulin and C-peptide concentrations. Exploratory outcomes included norepinephrine, growth hormone, and bone homeostatic markers carboxy-terminal collagen crosslinks (CTX) and procollagen type I amino-terminal propeptide (PINP).

**Results:**

During GLP-2 infusions, steady-state plasma GLP-2 concentrations were 50-fold higher than during placebo. Compared to placebo, GLP-2 increased glucagon secretion slightly during euglycemia, and not during insulin-induced hypoglycemia or hyperglycemia. Ex vivo, GLP-2 did not affect glucagon secretion from isolated human islets. GLP-2 did not affect circulating concentrations of insulin, C-peptide, growth hormone, norepinephrine, or CTX during hypoglycemia, euglycemia, or hyperglycemia. GLP-2 decreased PINP during euglycemia and hyperglycemia.

**Conclusion:**

Exogenous GLP-2 increased glucagon secretion slightly during euglycemia and not during insulin-induced hypoglycemia or hyperglycemia in healthy young men.

The pancreatic hormones insulin and glucagon play central roles in balancing blood glucose concentrations [1]. Insulin, secreted postprandially from pancreatic β cells, lowers plasma glucose, whereas glucagon, secreted during fasting from pancreatic α cells, increases plasma glucose [1]. Several gut-derived hormones affect the regulation of insulin and glucagon secretion. Glucagon-like peptide 1 (GLP-1), secreted from enteroendocrine L cells, and glucose-dependent insulinotropic polypeptide (GIP), secreted from enteroendocrine K cells, are well recognized as incretin hormones potentiating glucose-stimulated insulin secretion from pancreatic β cells [2-5]. In addition, both GLP-1 and GIP exert effects on pancreatic α cells; with GIP stimulating glucagon secretion during normal to low plasma glucose concentrations [6, 7] and GLP-1 inhibiting secretion during high plasma glucose concentrations [8-10]. Glucagon-like peptide 2 (GLP-2), often referred to as GLP-1's “sister hormone,” is a 33-amino acid peptide hormone derived from the same precursor, proglucagon, and cosecreted with GLP-1 in equimolar quantities from enteroendocrine L cells [11, 12]. GLP-2 is mostly recognized for its intestinotrophic effect used in the treatment of short bowel syndrome [13-16]. Interestingly, exogenous GLP-2 has been reported to increase glucagon levels during euglycemia and hyperglycemia in the isolated rat pancreas [17] and in humans [18-21], supposedly via direct stimulation of GLP-2 receptors on pancreatic α cells [17]. Conversely, in an extensive study in mice, GLP-2 was unable to stimulate glucagon secretion from perfused pancreatic islets in vitro or during insulin-induced hypoglycemia in vivo [22]. Thus, the role of GLP-2 in glucose metabolism remains unclear. GLP-2 has also been implicated in the regulation of bone homeostasis [23-27] as exogenous GLP-2 seems to reduce bone resorption as assessed by the circulating bone resorption marker carboxy-terminal collagen crosslinks (CTX) [24, 25, 27-31].

Here, using a specific and sensitive glucagon sandwich enzyme-linked immunosorbent assay (ELISA), we reevaluated the effect of exogenous GLP-2 on plasma glucagon levels during hypoglycemia, euglycemia, and hyperglycemia in lean, healthy, young men. We also evaluated, in an exploratory manner, the glucose-dependent effects of exogenous GLP-2 on circulating insulin, C-peptide, norepinephrine, growth hormone, the bone resorption marker CTX, and the bone formation marker procollagen type I N-terminal propeptide (PINP). Lastly, we investigated the direct effects of GLP-2 on glucagon secretion at different glucose concentrations in isolated human islets.

## Materials and Methods

### Approvals and Registrations

This study was performed at the Center for Clinical Metabolic Research, Gentofte Hospital, University of Copenhagen, Denmark, after approval by the scientific-ethics committee of the Capital Region of Denmark (record No. H-18046965) and the Danish Data Protection Agency. The study was registered at ClinicalTrials.gov (ID: NCT03954873) and was performed in accordance with the Helsinki Declaration II.

### Study Participants

Eligible study participants were White men aged 18 to 40 years with a body mass index (BMI) of 18.5 to 24.9, fasting blood glucose of 6 mmol/L or less, glycated hemoglobin A_1c_ (HbA_1c_) less than 42 mmol/mol (6.0%), and hemoglobin within normal range. Exclusion criteria included intestinal disease, liver disease and/or plasma alanine transaminase more than 2 times the upper limit of normal values, bilirubin greater than 25 µmol/L, reduced kidney function (estimated glomerular filtration rate <60 mL/min/1.73 m^2^), active or recent malignant disease, treatment that could not be paused for 12 hours, and first-degree relatives with diabetes.

### Study Design and Clinical Experimental Procedures

The study was designed as a randomized, double-blind, placebo-controlled, crossover study. All participants underwent a screening visit for eligibility followed by 6 study days (performed in a randomized order and separated by at least 7 days): 2 study days involving a hyperinsulinemic hypoglycemic clamp, 2 study days with plasma glucose at fasting levels, and 2 study days involving a hyperglycemic clamp. During each clamp condition, participants received a double-blinded intravenous (i.v.) infusion with placebo and GLP-2, respectively. The primary end point was GLP-2–induced change in baseline-subtracted area under the curve (bsAUC) for glucagon concentrations during hypoglycemia, euglycemia, and hyperglycemia, respectively. Study participants arrived at the clinical research facility after having fasted overnight (for ≥10 hours including water, coffee, prescription and nonprescription medication, and tobacco) and being instructed to abstain from strenuous physical activity or consumption of alcohol 48 hours up to the study day. Cannulas were inserted in the cubital veins; one was used for the collection of blood samples, and one in the contralateral vein was used for i.v. infusions. The forearm, from which blood samples were drawn, was wrapped in a heating pad for arterialization of venous blood. The infusion with either GLP-2 or isotonic saline (placebo) was started at time −10 minutes at a GLP-2 infusion rate of 6 pmol/kg/min (or an equal volume of isotonic saline) to reach the steady-state concentration and then reduced to 2 pmol/kg/min (or an equal volume of isotonic saline) from time 0 to 90 minutes to maintain steady state. Plasma glucose was measured bedside every 5 minutes, allowing the plasma glucose level to be clamped using an adjustable i.v. infusion of 20% glucose (w/v). On hypoglycemic study days, a fixed continuous i.v. infusion with insulin (1.5 mU/kg/min) was started at time 0 minutes, and plasma glucose was clamped at approximately 2.5 mmol/L during time 30 to 60 minutes. At time 60 minutes, the insulin infusion was stopped, and during time 60 to 90 minutes, the recovery from hypoglycemia was evaluated (during this period glucose was infused only if plasma glucose dropped to <2.5 mmol/L). On euglycemic study days, plasma glucose remained at baseline (euglycemic) levels (∼5 mmol/L). On hyperglycemic study days, a bolus infusion of 20% (w/v) glucose (volume calculated as previously described [6]) was infused at time 0 minutes, and then plasma glucose was clamped at approximately 10 mmol/L during time 0 to 90 minutes.

On all study days, blood samples were collected at time −20, −10, 0, 10, 20, 30, 40, 50, 60, 75, and 90 minutes. For measurement of plasma glucose, blood was sampled into sodium-fluoride tubes and centrifuged immediately at 7400*g* at room temperature for 30 seconds before bedside plasma analysis using the glucose oxidase method (Yellow Spring Instruments 2900 STAT glucose analyzer, Xylem Inc). For analysis of GLP-2 and glucagon, blood was collected in chilled tubes (on ice) containing EDTA and a specific dipeptidyl peptidase 4 inhibitor (1 mmol/L valine-pyrrolidine). For analysis of insulin, C-peptide, growth hormone, norepinephrine, PINP, and CTX, blood was sampled in plain tubes and left for coagulation at room temperature for 10 minutes. All tubes were then centrifuged for 15 minutes at 2000*g* and 4 °C, and plasma/serum was distributed to storage tubes and stored at −20 °C (−80 °C for insulin and C-peptide) until batch analysis. Blood pressure and heart rate were measured at time −10, 30, 60, and 90 minutes.

### Laboratory Methods

For plasma measurements of GLP-2, plasma was extracted in a final concentration of 75% ethanol before analysis. Intact GLP-2 was measured as previously described (Jens Juul Holst–University of Copenhagen catalog No. 92160, RRID:AB_2943622) [11]. Plasma glucagon was measured by a sandwich ELISA (Mercodia catalog No. 10-1271-01, RRID:AB_2737304) [32]. Serum insulin and C-peptide concentrations were measured with a 2-sided sandwich immunoassay using direct chemiluminescent technology (Siemens catalog No. 10995628, RRID:AB_2941780 [insulin] and catalog No. 10995541, RRID:AB_2941781 [C-peptide]). CTX, PINP, and growth hormone were measured with IDS-iSYS assays (Immunodiagnostic Systems catalog No. IS-3000, RRID:AB_2910613 [CTX], catalog No. IS-4000, RRID:AB_2936077 [PINP], and catalog No. IS-3700, RRID:AB_2861356 [growth hormone]). Plasma norepinephrine was measured using an ELISA (Labor Diagnostika Nord catalog No. BA E-5200R, RRID:AB_2909484).

### Peptides

Synthetic human GLP-2 (1-33) was purchased from Bachem Laboratories and was demonstrated to be more than 97% pure and identical to the natural human peptide by high-performance liquid chromatography and sequence and mass analysis by Bachem Laboratories. GLP-2 powder was dissolved in a solution of 0.9% NaCl, 0.5% human serum albumin, and NaHCO_3_, the latter added to provide a pH of 8.5 to enhance the solubility of the GLP-2. The solution was subjected to sterile filtration and tested for sterility and endotoxins by the Capital Region Pharmacy, dispensed into glass vials, and stored at −20 °C until use. An uninvolved laboratory technician prepared the infusions, keeping the contents unknown to the primary investigator and the study participant. On GLP-2 study days, a glass vial of GLP-2 was thawed, and appropriate amounts (based on the participant's body weight) were injected into an infusion bag containing 250-mL sterile water with 0.9% NaCl and 0.5% human serum albumin. On placebo study days, a bag containing 250-mL sterile water with 0.9% NaCl and 0.5% human serum albumin was infused.

### Human Pancreatic Islets—Experimental Procedures and Analyses

Isolated pancreatic islets from a total of 3 cadaveric nondiabetic donors (Table 1) were obtained from Prodo Laboratories and maintained in F10 Nutrient Mixture 1X + Glutamax medium supplemented with 10% fetal bovine serum and 1% penicillin and streptomycin (all Gibco) until glucose-dependent secretion assays. Briefly, isolated islets were handpicked and washed twice in Krebs Ringer HEPES buffer (KRHB; 115-mmol/L sodium chloride, 4.7-mmol/L potassium chloride, 2.6-mmol/L calcium chloride, 1.2-mmol/L potassium phosphate, 1.2-mmol/L magnesium sulfate·7H_2_O, 5-mmol/L sodium bicarbonate, 20-mmol/L HEPES, 2 mg/mL bovine serum albumin) with 5.5-mmol/L glucose before sequential 30 minutes in incubations in KRHB with or without GLP-2 (same as used for the clinical study). Initial incubations for the glucose-dependent assays were all 5.5 mmol/L glucose followed by either 2, 5.5, or 11 mmol/L glucose. Each experiment used islets from a single donor, and experimental conditions were performed in technical duplicate (groups of 25-30 islets). Secreted glucagon was measured using Human Glucagon ELISA (Mercodia catalog No. 10-1271-01, RRID:AB_2737304) and normalized to double-stranded DNA content to account for variations in islet size using the QuantiFlour dsDNA system (Promega) according to the manufacturer's instructions.

**Table 1.** Islet donor details

Variable	Donor 1	Donor 2	Donor 3
Sex, male/female	Male	Male	Male
Age, y	47	58	48
Height, m	1.75	1.64	1.78
Weight, kg	108	72	70
BMI	35.3	26.8	22.1
HbA_1c_, %	5.5	4.9	5.3
History of diabetes, yes/no	No	No	No
Cause of death	Stroke	Stroke	Head trauma

Clinical characteristics for the 3 islet donors.

Abbreviations: BMI, body mass index; HbA_1c_, glycated hemoglobin A_1c_.

### Calculations and Statistical Analyses

Based on an SD of the primary end point (bsAUC for glucagon) from a previous study with similar methodology [18], a power of 80%, and a level of statistical significance of 5%, we calculated the number of study participants to be 8.4 to detect a minimal relevant difference of 15% in bsAUC for glucagon. To ensure sufficient power, 10 study participants were included. Results are reported as mean and SD unless otherwise stated. Calculation of bsAUC was based on the trapezoidal rule. For GLP-2, glucagon, insulin, and C-peptide, baseline values were defined as the mean of time −20 and −10 minutes. For plasma glucose, CTX, PINP, growth hormone, and norepinephrine, baseline values were defined as the first measurement. Total glucose infusion was evaluated as cumulated glucose per kilogram of body weight at 90 minutes. Heart rate and blood pressure were evaluated as mean values. End points were assumed to be following a normal distribution, and paired *t* tests were used to test for statistically significant differences between end points. Due to the explorative nature of the study, reported *P* values were not adjusted for multiple testing, and thus, should be interpreted accordingly. As recommended by the CONSORT 2025 guidelines for reporting on randomized controlled trials [33], significance testing of baseline values was not performed. Statistical analyses were performed using GraphPad Prism 10.1.2 (GraphPad Software). A 2-sided *P* value of less than .05 was used as the statistical significance level. For in vitro islet experiments, a repeated-measures 2-way analysis of variance with Šídák's multiple comparisons test was used to compare GLP-2–treated and untreated control groups. Statistical significance between groups was determined by a *P* value of less than .05.

## Results

### Study Participants

Ten healthy men with a median (interquartile range) age of 22 (21-23) years, BMI of 23.5 (23.3-23.8), and HbA_1c_ of 4.8 (4.6-5.1)% (29 [26.5-32.5] mmol/mol), were included after screening, during which oral and written informed consents were obtained. Participant characteristics are provided in Table 2. All 10 study participants completed the 6 study days.

**Table 2.** Baseline characteristics

Variable	Median (interquartile range)
Sex, male/female	10/0
Age, y	22 (21-23)
Height, m	1.84 (1.77-1.92)
Weight, kg	79 (70-86)
BMI	23.5 (23.3-23.8)
Waist/hip ratio	0.89 (0.86-0.92)
Systolic bp, mm Hg	126 (117-130)
Diastolic bp, mm Hg	78 (65-79)
Heart rate, bpm	76 (66-83)
HbA_1c_, %	4.8 (4.6-5.1)
HbA_1c_, mmol/mol	29.0 (26.5-32.5)

Data are shown as median with interquartile range in parentheses.

Abbreviations: BMI, body mass index; bp, blood pressure; bpm, beats per minute; HbA_1c,_ glycated hemoglobin A_1c_.

### Supraphysiological Plasma Glucagon-Like Peptide 2 (GLP-2) Concentrations During GLP-2 Infusion

**Figure 1.**
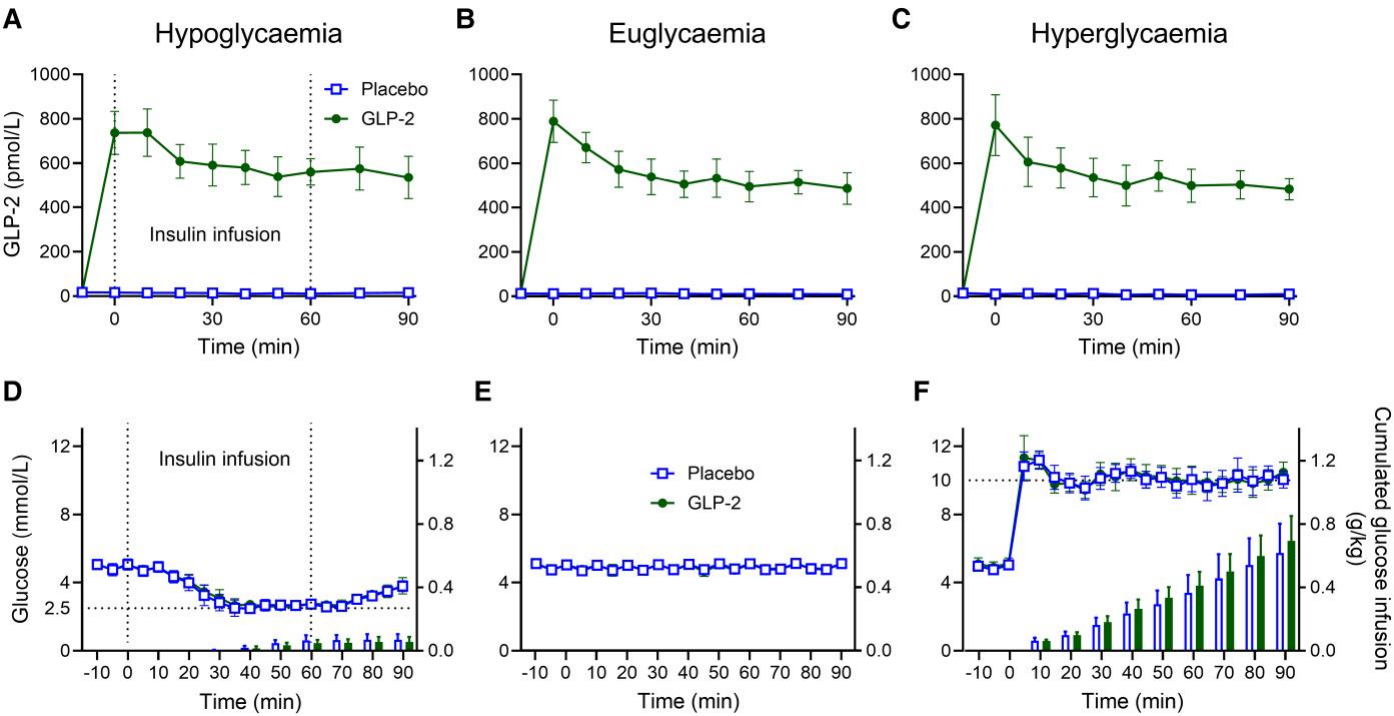
Plasma GLP-2, plasma glucose, and cumulated amount of glucose infused. A to C, Plasma GLP-2, and D to F, plasma glucose levels (lines) with cumulated glucose infused (bars) in 10 healthy male participants during a total of 6 study days (2 days of hypoglycemia, euglycemia, and hyperglycemia, respectively) with continuous i.v. infusions of placebo (saline) (open square) and GLP-2 (closed circle), respectively. Dotted vertical lines denote the start/end of insulin infusion. Data were compared by paired *t* tests. Statistically significant differences (*P* < .05) are marked with an asterisk (*). Data are presented as mean ± SD. GLP-2, glucagon-like peptide 2; i.v., intravenous.

Steady-state GLP-2 levels during hypoglycemia, euglycemia, and hyperglycemia were similar during GLP-2 infusions (560 ± 110, 510 ± 93, and 511 ± 93 pmol/L) and placebo infusions (13 ± 7.1, 11 ± 3.4, and 9.0 ± 3.2 pmol/L), respectively (Fig. 1A-1C, Table 3).

**Table 3.** Plasma/serum measurements

	Hypoglycemia			Euglycemia			Hyperglycemia		
	Placebo	GLP-2	Mean difference (95% CI)	Placebo	GLP-2	Mean difference (95% CI)	Placebo	GLP-2	Mean difference (95% CI)
**GLP-2**									
Baseline, pmol/L	17.4 ± 7.1	16.7 ± 7.8	−0.7 (−7.2 to 5.8)	12.7 ± 5.5	18.5 ± 16.3	5.9 (−7.3 to 19.0)	14.9 ± 5.7	18.4 ± 4.9	3.5 (−2.1 to 9.1)
bsAUC_-10-90min_, nmol/L × min	−0.4 ± 0.5	55.7 ± 9.8	56.1 (49.1 to 63.1)^a^	−0.1 ± 0.4	51.7 ± 8.8	51.8 (45.4 to 58.3)*^a^*	−0.5 ± 0.5	50.6 ± 10.3	51.1 (43.6 to 58.6)*^a^*
Steady state_30-90min_, pmol/L	12.8 ± 7.1	560.3 ± 109.7	547.5 (470.6 to 624.4)*^a^*	11.3 ± 3.4	510.1 ± 92.9	498.8 (432.8 to 564.8)*^a^*	9.0 ± 3.2	510.6 ± 93.1	501.6 (435.0 to 568.2)*^a^*
**Plasma glucose**									
Baseline, mmol/L	5.0 ± 0.3	5.1 ± 0.3	0.08 (−0.2 to 0.3)	5.1 ± 0.2	5.1 ± 0.2	−0.01 (−0.2 to 0.2)	5.1 ± 0.2	5.2 ± 0.3	0.2 (−0.1 to 0.4)
Steady state*^b^*, mmol/L	2.7 ± 0.2	2.7 ± 0.1	0.1 (−0.03 to 0.2)	4.9 ± 0.2	4.9 ± 0.2	−0.001 (−0.1 to 0.1)	10.2 ± 0.2	10.2 ± 0.2	0.03 (−0.1 to 0.1)
**Glucose infusion**									
Time 0-90 min, g/kg	0.07 ± 0.04	0.05 ± 0.03	−0.01 (−0.04 to 0.01)	0	0	—	0.62 ± 0.19	0.69 ± 0.16	0.08 (0.003 to 0.15)*^a^*
**Glucagon**									
Baseline, pmol/L	7.5 ± 2.9	8.5 ± 2.5	1.0 (−2.0 to 4.0)	7.6 ± 2.8	5.8 ± 2.9	−1.8 (−4.4 to 0.8)	7.6 ± 2.6	8.7 ± 3.7	1.0 (−1.9 to 4.1)
bsAUC_−10-90min_, pmol/L × min	1243 ± 533	1406 ± 350	163 (−269 to 596)	*−41* ± 166	306 ± 197	347 (164 to 530)*^a^*	*−*488 ± 196	*−*485 ± 286	3 (−210 to 216)
**Insulin**									
Baseline, pmol/L	45.1 ± 37.6	43.5 ± 11.9	−1.6 (−27.0 to 23.8)	44.7 ± 23.6	42.2 ± 16.5	−2.5 (−10.3 to 5.3)	41.3 ± 18.9	44.3 ± 18.3	3.0 (−5.4 to 11.4)
bsAUC_−10-90min_, nmol/L × min	28.8 ± 2.8	24.4 ± 4.1	−4.4 (−6.2 to −2.6)*^a^*	−0.8 ± 1.4	0.2 ± 0.8	1.0 (−0.1 to 2.0)	16.3 ± 11.2	19.7 ± 10.3	3.4 (−1.4 to 8.2)
**C-peptide**									
Baseline, pmol/L	338.6 ± 181	336.5 ± 95.1	−2.1 (−93.7 to 89.5)	331.1 ± 133.3	324.4 ± 112.8	−6.7 (−40.6 to 27.2)	331.3 ± 123.3	345.1 ± 142.9	13.8 (−34.1 to 61.6)
bsAUC_−10-90min_, nmol/L × min	−16.4 ± 10.1	−15.8 ± 5.0	0.7 (−5.2 to 6.6)	−2.8 ± 3.2	−2.1 ± 2.0	0.7 (−1.9 to 3.4)	78.5 ± 29.3	83.2 ± 31.1	4.8 (−7.9 to 17.4)
**Growth hormone**									
Baseline, pg/mL	1.6 ± 2.0	2.3 ± 4.4	0.7 (−1.2 to 2.6)	2.1 ± 2.8	2.4 ± 3.2	0.2 (−1.5 to 2.0)	2.6 ± 4.9	2.0 ± 3.1	−0.6 (−3.1 to 2.0)
bsAUC_−10-90min_, pg/mL × min	569 ± 505	330 ± 479	−239 (−486 to 8)	−100 ± 214	−65 ± 259	35 (−94 to 163)	−137 ± 325	−130 ± 249	7 (−163 to 177)
**Norepinephrine**									
Baseline, ng/mL	0.18 ± 0.07	0.15 ± 0.07	−0.03 (−0.09 to 0.03)	0.15 ± 0.08	0.15 ± 0.05	0.002 (−0.04 to 0.04)			
bsAUC_−10-90min_, ng/mL × min	4.3 ± 6.6	8.8 ± 5.6	4.5 (−0.2 to 9.1)	2.0 ± 3.9	2.2 ± 3.1	0.2 (−3.8 to 4.3)			
**CTX**									
Baseline, ng/mL	0.7 ± 0.3	0.7 ± 0.3	0.02 (−0.1 to 0.1)	0.6 ± 0.2	0.7 ± 0.3	0.07 (−0.05 to 0.2)	0.7 ± 0.3	0.7 ± 0.3	0.01 (−0.1 to 0.1)
bsAUC_−10-90min_, ng/mL × min	−14.5 ± 8.3	−14.7 ± 6.6	−0.1 (−5.3 to 5.0)	−5.2 ± 9.3	−9.2 ± 3.5	−4.1 (−10.5 to 2.3)	−15.9 ± 6.1	−15.1 ± 9.9	0.8 (−6.0 to 7.6)
**PINP**									
Baseline, ng/mL	90.4 ± 34.8	82.7 ± 21.2	−7.7 (−25.9 to 10.5)	90.5 ± 28.5	90.8 ± 24.3	0.2 (−9.1 to 9.6)	90.9 ± 27.5	90.2 ± 24.9	−0.7 (−10.5 to 9.1)
bsAUC_−10-90min_, ng/mL × min	−1050 ± 840	−1023 ± 493	27 (−485 to 539)	141 ± 304	−325 ± 256	−466 (−727 to −206)*^a^*	−154 ± 387	−718 ± 387	*−*564 (−825 to −303)*^a^*

Overview of plasma and serum measurements from the 6 study days. Data are presented as mean ± SD and mean difference with 95% CIs. Results are compared by paired *t* tests.

Abbreviations: bsAUC, baseline-subtracted area under the curve; CTX, carboxy-terminal collagen crosslinks; GLP-2, glucagon-like peptide 2; PINP, procollagen type I amino-terminal propeptide.

^*a*^ *P* less than .05 when comparing GLP-2 to placebo during the same glycemic level.

^*b*^Steady state is based on time −20 to 90 minutes for euglycemia, time 5 to 90 minutes for hyperglycemia, and time 30 to 60 minutes for hypoglycemia (see Fig. 1D-1F).

### Successful Clamping of Plasma Glucose at Hypoglycemic, Euglycemic, and Hyperglycemic Levels

Steady-state plasma glucose levels during hypoglycemia were obtained at time 30 to 60 minutes (Fig. 1D, lines), with a mean of 2.7 ± 0.2 and 2.7 ± 0.1 mmol/L (*P* = .15) during placebo and GLP-2 infusion, respectively. No difference in cumulated amount of glucose infused during hypoglycemia was observed at time 60 minutes when insulin infusion was stopped (*P* = .070) or at time 90 minutes (*P* = .23) (Fig. 1D, bars). Steady-state plasma glucose concentrations during euglycemia (time −20 to 90 minutes) (Fig. 1E, lines) were 4.9 ± 0.2 and 4.9 ± 0.2 mmol/L (*P* = .98) during placebo and GLP-2 infusion, respectively; no glucose or insulin was infused on either of the 2 experiments at euglycemia (Fig. 1E). Steady-state plasma glucose during hyperglycemia was obtained at time 5-90 minutes (Fig. 1F, lines) with a mean of 10.2 ± 0.2 and 10.2 ± 0.2 mmol/L (*P* = .57) during placebo and GLP-2 infusion, respectively. Slightly more glucose was needed to maintain hyperglycemia during GLP-2 infusion compared to during placebo infusion (0.69 ± 0.16 vs 0.62 ± 0.19 g glucose/kg, *P* = .044) (see Fig. 1F, bars, and Table 3).

### Exogenous Glucagon-Like Peptide 2 Increased Plasma Glucagon Slightly During Euglycemia, but Not During Hypoglycemia or Hyperglycemia

**Figure 2.**
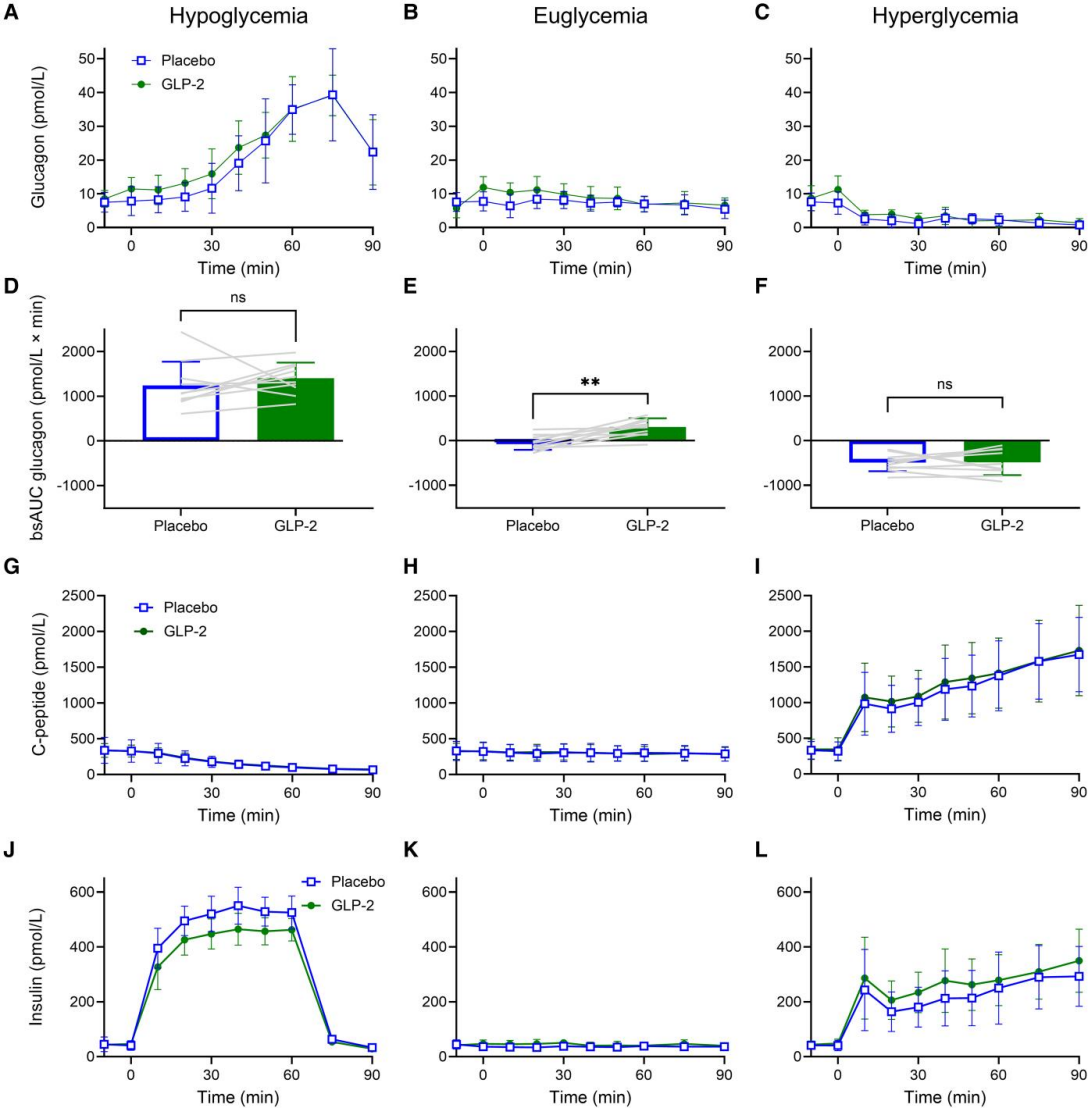
Glucagon, C-peptide, and insulin. A to C, Plasma glucagon; D to F, bsAUC glucagon; G to I, serum C-peptide; and J to L, serum insulin in 10 healthy male participants during a total of 6 study days (2 days of hypoglycemia, euglycemia, and hyperglycemia, respectively) with continuous i.v. infusions of placebo (saline) (open square) and GLP-2 (closed circle), respectively. BsAUC data were compared by paired *t* tests. Asterisks (*) mark statistically significant differences, (*P < .05; **P < .005). Data are presented as mean ± SD. bsAUC, baseline-subtracted area under the curve; GLP-2, glucagon-like peptide 2; i.v., intravenous.

Baseline glucagon concentrations were similar on all 6 study days (see Table 3). GLP-2 increased bsAUC for plasma glucagon during euglycemia (−41 ± 166 [placebo] vs 306 ± 197 pmol/L × min [GLP-2]; *P* < .005), but did not significantly affect bsAUC for plasma glucagon during hypoglycemia (1243 ± 533 [placebo] vs 1406 ± 350 pmol/L × min [GLP-2]), or hyperglycemia (−488 ± 196 [placebo] vs −485 ± 286 pmol/L × min [GLP-2]) (Fig. 2D-2F, Table 3). However, during hypoglycemia, a trend toward increased bsAUC of glucagon during GLP-2 infusion was seen, with only 2 participants showing a higher bsAUC during placebo (see Fig. 2D). No statistically significant differences were seen between GLP-2 and placebo during the recovery from hypoglycemia (time 60-90 minutes) (see Fig. 2A).

### Exogenous Glucagon-Like Peptide 2 Did Not Affect C-Peptide but Slightly Reduced Insulin Levels During Hypoglycemia

There were no differences in baseline C-peptide, baseline insulin, or bsAUC for C-peptide between GLP-2 and placebo study days during hypoglycemia, euglycemia, or hyperglycemia (Fig. 2G-2L, Table 3). BsAUC for insulin during insulin-induced hypoglycemia was slightly but significantly lower during GLP-2 infusion compared to placebo (28.8 ± 2.8 [placebo] vs 24.4 ± 4.1 nmol/L × min [GLP-2]; *P*  **<** .005) (see Fig. 2J, Table 3). During euglycemia and hyperglycemia, bsAUC for insulin was similar during placebo and GLP-2 infusion (see Fig. 2K and 2L, Table 3).

### Growth Hormone and Norepinephrine Were Not Affected by Exogenous Glucagon-Like Peptide 2

**Figure 3.**
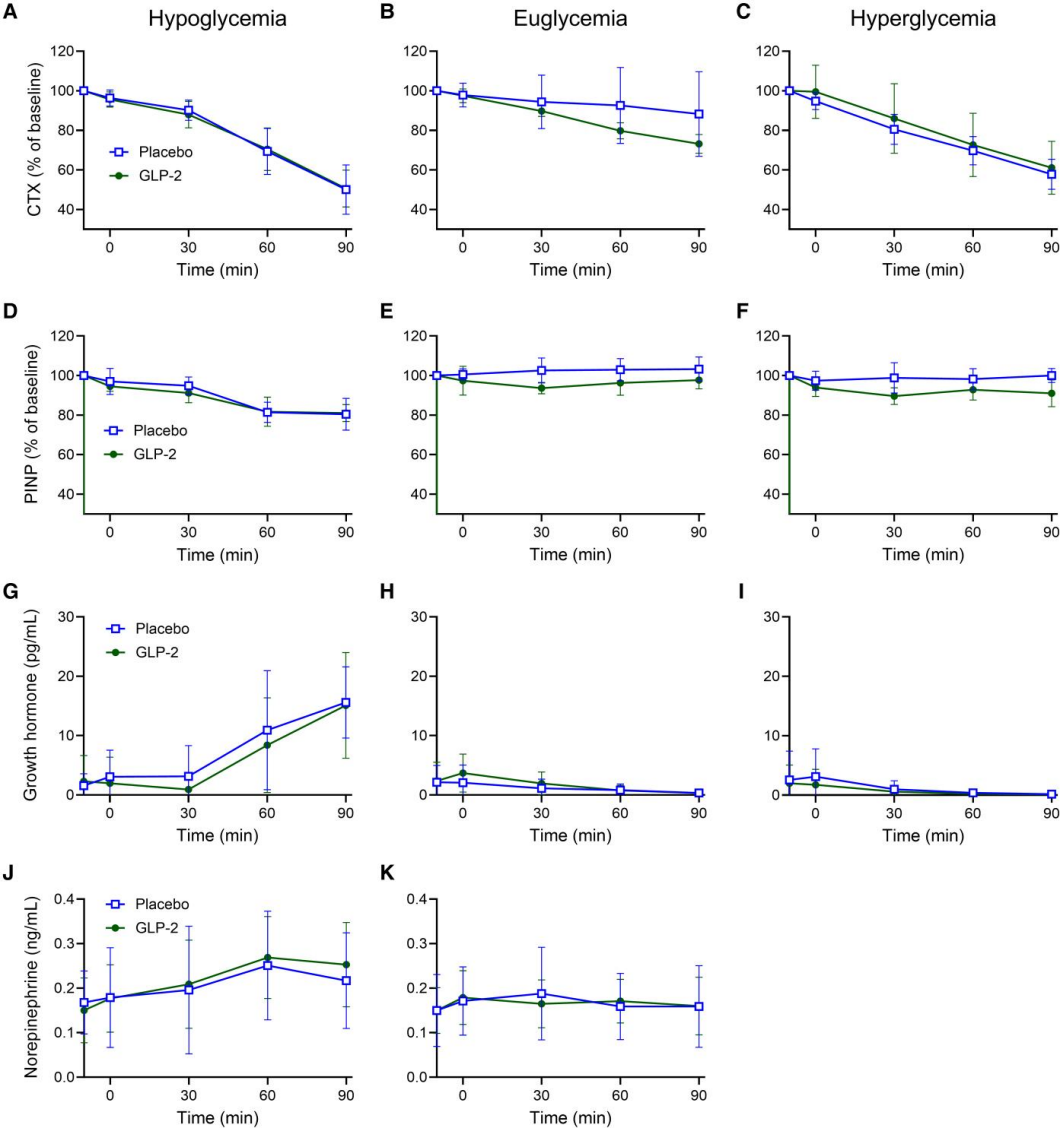
CTX, PINP, growth hormone, and norepinephrine. A to C, Serum CTX; D to F, PINP; G to I, growth hormone; and J and K, norepinephrine in 10 healthy male participants during a total of 6 study days (2 days of hypoglycemia, euglycemia, and hyperglycemia, respectively) with continuous i.v. infusions of placebo (saline) (open square) and GLP-2 (closed circle), respectively. Norepinephrine was not measured during hyperglycemia. Data are presented as mean ± SD. CTX, carboxy-terminal collagen crosslinks; GLP-2, glucagon-like peptide 2; i.v., intravenous; PINP, procollagen type 1 amino-terminal propeptide.

Baseline levels of counterregulatory hormones growth hormone and norepinephrine were similar across all interventions. Compared to placebo, the bsAUC for either hormone was not affected by exogenous GLP-2 (Fig. 3G-3K, Table 3).

### Procollagen Type I Amino-Terminal Propeptide Was Reduced by Exogenous Glucagon-Like Peptide 2 During Euglycemia and Hyperglycemia

No differences in baseline CTX or bsAUC for CTX were observed between GLP-2 infusion and placebo infusion during hypoglycemia, euglycemia, or hyperglycemia (Fig. 3A-3C, Table 3). However, a nonstatistically significant decrease in CTX was observed during euglycemia (Fig. 3B). Baseline PINP concentrations were similar during GLP-2 infusion and placebo infusion during all 3 glycemic levels. Compared to placebo, GLP-2 infusion significantly reduced bsAUC for PINP during euglycemia (141 ± 304 [placebo] vs −325 ± 256 ng/mL × min [GLP-2]; *P* = .003) and hyperglycemia (−154 ± 387 [placebo] vs −718 ± 387 ng/mL × min [GLP-2]; *P* = .0009) (Fig. 3E and 3F, Table 3). No statistically significant difference between GLP-2 infusion and placebo was found in bsAUC for PINP during hypoglycemia (Fig. 3D, Table 3).

### Heart Rate Was Slightly Raised by Exogenous Glucagon-Like Peptide 2 During Hypoglycemia

There were no differences between study days for baseline or mean values for systolic and diastolic blood pressure, nor for heart rate during euglycemia and hyperglycemia (data not shown). However, heart rate during hypoglycemia was slightly but statistically significantly higher during GLP-2 infusion compared to placebo (61.1 ± 6.7 [placebo] vs 64.1 ± 6.1 bpm [GLP-2]; *P* = .048)

### Glucagon-Like Peptide 2 Did Not Affect Glucagon Secretion From Isolated Human Islets

**Figure 4.**
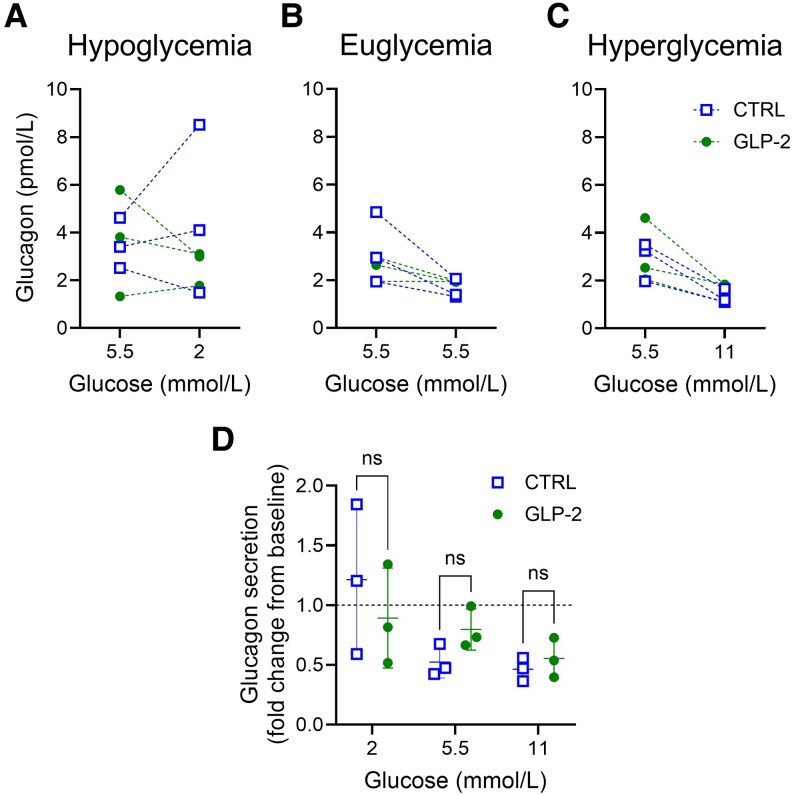
Ex vivo human islet studies. Glucagon secretion from isolated human islets with (closed circle) or without (open square) 2.5 nmol/L GLP-2 on changing glucose from euglycemia (5.5 mmol/L) to A, hypoglycemia (2.0 mmol/L glucose); B, euglycemia (5.5 mmol/L), or C, hyperglycemia (11.0 mmol/L glucose). Data points represent the mean of technical duplicates from 3 individual nondiabetic donors. Differences between GLP-2 and control groups on challenge were not statistically significant both pre challenge (*P* > .99, *P* = .51, and *P* = .92, respectively) and on challenge (*P* = .27, *P* = .81, and *P* = .79 for 2.0, 5.5, and 11.0 mmol/L glucose, respectively) using D, random-measures 2-way analysis of variance with Šídák's multiple comparisons test. CTRL, control; GLP-2, glucagon-like peptide 2.

In sequential glucose-dependent glucagon secretion experiments with isolated human islets designed to mimic the clinical study design, GLP-2 did not affect glucagon secretion during euglycemia (5.5 mmol/L) or at subsequent hypoglycemia (2 mmol/L glucose), euglycemia, or hyperglycemia (11 mmol/L glucose) (Fig. 4A-4D).

## Discussion

Here, using a sensitive and specific glucagon assay, we show that exogenous GLP-2, compared to placebo, increases glucagon secretion only slightly during euglycemia, and not during hyperglycemia or insulin-induced hypoglycemia in healthy men.

Our focus on the glucagonotropic effects of gut-derived hormones originates from an interest in the physiological interaction between the gut and pancreas and from the potential therapeutic use of glucagonotropic hormones as safeguards against hypoglycemia. Furthermore, as GLP-2 receptor agonists are used in the treatment of short bowel syndrome [34], and dual GLP-1/GLP-2 agonists are being investigated as potential weight loss therapies (NCT05788601) [35, 36], the glucometabolic effects of exogenous GLP-2 have gained renewed interest.

We used a GLP-2 infusion rate of 6 pmol/kg/min for 10 minutes, followed by 2 pmol/kg/min based on the design of a previous study [18]. Despite reaching steady-state concentrations of GLP-2 at around 10 times physiological levels, we observed only a small increase in glucagon during euglycemia and a nonstatistically significant trend during hypoglycemia, suggesting that GLP-2 has no physiologically relevant effect on glucagon secretion—at least under the tested conditions. Additionally, as the small increases in glucagon did not affect glucose levels during euglycemia or the amount of glucose required to maintain the hypoglycemic clamp, it may be of limited clinical relevance.

Previous studies have shown robust glucagonotropic effects of exogenous GLP-2 in healthy volunteers [18, 20], presumably via GLP-2 receptors on pancreatic α cells [17]. The discrepancy in magnitude of glucagon response between the present and previous findings on GLP-2's glucagonotropic effect may relate to the different glucagon assays employed or the GLP-2 infusion rate used. In the present study, we used a highly specific and sensitive sandwich ELISA, whereas previous studies showing robust glucagonotropic effects of exogenous GLP-2 have used a glucagon radioimmunoassay, which may cross-react with GLP-2 at high concentrations. In support of this notion, we observed no glucagon-derived effects on glucose, insulin, or C-peptide during GLP-2 infusion. Thus, previous studies may have overestimated the glucagonotropic effect of GLP-2. During insulin-induced hypoglycemia, GLP-2 did not compromise nor potentiate the counterregulatory glucagon response or responses of the counterregulatory hormones norepinephrine and growth hormone.

Our ex vivo data, using GLP-2 concentrations approximately 5 times higher than our clinical study, showed no GLP-2–induced glucagon secretion from isolated human islets exposed to glucose concentrations reflecting hypoglycemia, euglycemia, and hyperglycemia, respectively, suggesting no direct glucagonotropic effect of GLP-2 on human α cells, at least under the specific, tested experimental conditions. Our overall findings thus align with the results from Bahrami et al [22], who did not find any glucagonotropic effects of GLP-2 on isolated pancreatic islets or during insulin-induced hypoglycemia in vivo in mice. Although their results regarding exogenous GLP-2 were negative, GLP-2 receptor knockout in obese mice led to increased fasting and fed glucose levels, reduced β-cell mass, and paradoxically improved oral glucose tolerance. These effects were not observed in lean or diabetic mice, and since our study exclusively involved lean men, we were unable to elucidate if these findings extend to humans. Nonetheless, as GLP-2–based therapies are being explored for obesity, such “obesity-specific” effects may prove clinically relevant.

A slightly greater amount of glucose was required to maintain hyperglycemia during GLP-2 infusion compared to placebo. However, although statistically significant, the mean difference of 0.075 (95% CI, 0.003-0.15) g/kg could be explained by the trend toward higher insulin concentrations (bsAUC) on the GLP-2 day (mean difference: 3.4 [95% CI, −1.4 to 8.2] nmol/L × min). Conversely, a small, but statistically significant, difference was seen in bsAUC for insulin on the hypoglycemia days, with insulin being slightly higher during placebo compared to GLP-2. The reason for the opposing effects of GLP-2 on insulin concentrations during hyperglycemia and hypoglycemia is unclear. GLP-2 has been shown to increase splanchnic blood flow [37], and this could hypothetically lead to an increased hepatic breakdown of insulin. However, whether GLP-2's effects on blood flow are influenced by blood glucose levels is unknown. In this study we found a slightly increased heart rate during hypoglycemia with GLP-2 compared to placebo, but no other changes in hemodynamic parameters. Importantly, the person preparing the insulin infusion on the hypoglycemic days was blinded to the intervention, making a systematic difference in the amount of insulin infused unlikely.

We observed no statistically significant effect of GLP-2 on the circulating bone resorption marker CTX, which is in contrast to some studies showing modest suppression of CTX after GLP-2 administration [25, 27-31]. In these studies, GLP-2 was administered as a single subcutaneous bolus (in one study an i.v. bolus was used [28]), resulting in very high peak GLP-2 concentrations—at least 3 times higher than peak levels reached in our study. We observed a slight decrease in the serum bone formation marker PINP during euglycemia and hyperglycemia on days with GLP-2. Similar observations, albeit transient in nature, have been made in healthy participants, patients with type 2 diabetes, and patients with ileostomy [25, 27, 29].

This study is an explorative study on a relatively small and homogeneous group (ie, healthy young men), and thus, the extent to which our results apply for women, patients with diabetes or obesity, as well as other populations, should be carefully considered. Additionally, as this study is primarily powered to show a difference in bsAUC for glucagon, any negative findings regarding other exploratory end points should be interpreted with caution.

Our results may be taken as disappointing in terms of using GLP-2 physiology as a glucagonotropic safeguard against hypoglycemia. On the other hand, the lack of clinically relevant glucometabolic effects of exogenous GLP-2 can be perceived as an advantage in terms of off-target effects of current and future GLP-2–based drugs for the treatment of short bowel syndrome and obesity, respectively, until ongoing clinical trials providing longer-term safety data are completed.

## Conclusion

Collectively, our findings show that supraphysiological levels of GLP-2 slightly but significantly increase glucagon secretion during euglycemia and not during insulin-induced hypoglycemia or hyperglycemia, in lean, young, healthy men. The observed glucagonotropic effects of GLP-2 during euglycemia appear smaller than previously reported [18-20], and seem of limited clinical relevance.

## Data Availability

The datasets generated during and/or analyzed during the current study are not publicly available but are available from the corresponding author on reasonable request.

## Clinical Trial Information

ClinicalTrials.gov registration number (ID: NCT03954873) (registered May 15, 2019).

## Abbreviations

BMI: body mass index
bsAUC: baseline-subtracted area under the curve
CTX: carboxy-terminal collagen crosslinks
ELISA: enzyme-linked immunosorbent assay
GIP: glucose-dependent insulinotropic polypeptide
GLP-1: glucagon-like peptide 1
GLP-2: glucagon-like peptide 2
i.v.: intravenous
PINP: procollagen type I amino-terminal propeptide
